# Intaglio surface trueness of dentures bases fabricated with 3D printing vs. conventional workflow: a clinical study

**DOI:** 10.1186/s12903-024-04439-8

**Published:** 2024-06-08

**Authors:** Andrei-Bogdan Faur, Raul Nicolae Rotar, Anca Jivănescu

**Affiliations:** 1 Department of Prosthodontics, University of Medicine and Pharmacy “Victor Babes”, B-dul Revolutiei 1989, No. 9, Timisoara, 300580 Romania; 2 TADERP Research Center, Department of Prosthodontics, University of Medicine and Pharmacy “Victor Babes”, B-dul Revolutiei 1989, No. 9, Timisoara, 300580 Romania

**Keywords:** 3D-printed denture, Intraoral scanning, Trueness, CAD/CAM

## Abstract

The latest generation of intraoral scanners can record the prosthetic field with relative ease, high accuracy and comfort for the patient, and have enabled fully digital protocols for designing and manufacturing complete dentures. The present study aims to examine the intaglio surface trueness of 3D printed maxillary dentures produced by fully digital workflow in comparison with dentures produced by analogue clinical and laboratory prosthetic workflow. The edentulous maxillary arch of 15 patients was scanned with an intraoral scanner as well as the intaglio of the delivered conventional denture. The scan of the edentulous arch was imported into a dental design software to produce the denture base which was then 3D printed. The intaglio surface of the finished 3D printed denture bases was digitized and used to assess the trueness of the printed denture bases compared to the intaglio surface of the conventional dentures as well as performing a trueness comparison in relation to the scanned edentulous arches. The dataset (*n* = 30) was subjected to Kruskal-Wallis test analysis, the significance level being established at α = 0.05. The results of the study showed that the printed group displayed better trueness values with a median of 176.9 μm while the analogue group showed a median of 342 μm. Employing a fully digital workflow to produce 3D-printed denture bases yields a consistent and precise manufacturing method when accounting for the intaglio surface of the denture.

## Background

Edentulous patients are still numerous worldwide as a result of the increase in lifespan of the population [1, 2]. Usually, the treatment of these cases follows the conventional approach using impression materials, dental casts, and wax try-ins. These procedures have been proved to lead to great results, the main disadvantage being related to many appointments and corrections until the final treatment result is achieved [3–5].

Apart from fixed partial dentures, the progress of intraoral scanners and digital impressions has led to treatment options also for complete edentulism [6, 7]. Digital methods provide several potential advantages when compared to the conventional approach, including the possibility of evaluating the digital impression and making the required adjustments, reduced working time, patient comfort, and better fit. However, one essential aspect that dictates the success of a complete denture is the accurate recording of the periphery of the edentulous arches guided by both the clinician and the orofacial muscular system [8, 9].

There are a few factors that can influence the efficiency and accuracy of intraoral scanners related to both the clinician and the patient. Ambient lighting conditions, the cutting and rescanning of an area, the morphology of the edentulous ridges as well as the anatomy of the palate, scanning pattern or the presence of fluids can interfere with an accurate digital impression. Knowing all these variables is important in order to minimize the errors than can occur during the digital impression procedure [10–12].

Usually, the treatment steps may follow either a partial digital workflow with the scanning of a conventional impression or more recently, a fully digital approach. Also, the development of 3D printers and printing materials has made the workflow more predictable. However, apart from the additive methods of manufacturing a complete denture, subtractive techniques are also an option used for the treatment of edentulous patients [13–15].

The purpose of our study was to investigate the intaglio surface trueness of 3D printed maxillary dentures produced by a fully digital workflow. The difference in intaglio surface trueness between analogue and 3D printed denture bases in relation to the edentulous arch was also investigated. The null hypothesis of this study was that there would be no differences regarding the trueness of the intaglio surface of 3D printed maxillary denture bases and the intaglio surface of denture bases made by analogue protocol.

## Methods

A total of 15 patients with edentulous maxillary arches were selected from the prosthodontic clinic patients for this study. Eligibility criteria were limited to patients with edentulous maxillary arch who meet the criteria for class 1 or class 2 of the ACP Classification System for Complete Edentulism [16] and an age range of 50 to 80 years. The different situation of the lower arch was considered irrelevant as only the maxillary arch was analysed.

Informed consent from the patients regarding the treatment plan and study participation, alongside the Ethical Committee approval from CECS UMFVBT Nr. 03/2023, was obtained. Each clinical case has been assigned to a study code name to assure that personal information, clinical data, future digital information, and intraoral scans of the patients were confidential, anonymous, and randomly assessed within the study.

All the patients received a complete maxillary denture produced by following the conventional clinical and laboratory protocol. The conventional analogue protocol consisted in the following steps:

1. Initial consultation of the edentulous patient, and preliminary alginate impressions of the edentulous arches were taken.
2. Obtaining the preliminary gypsum models and the custom impression trays;
3. Obtaining the final impressions with mucostatic technique by taking the following steps: A wax spacer with a uniform thickness of 2 mm was used on the preliminary models and the custom impression trays were designed by using a light-cured resin material (Palatray XL, Heraeus, Kulzer). A carbide bur was used to create perforations in order to improve retention of the impression material as well as applying a thin layer of adhesive (Tray Adhesive, DMG) to the internal surface. The final impressions were produced by implying a mucostatic technique, using a light bodied VPS impression material (Virtual, Ivoclar, Vivadent);
4. Producing the master cast and the occlusion rims;
5. Recording the intermaxillary relationships;
6. Mounting the anterior and posterior teeth and obtaining the try-in;
7. Aesthetic, phonetic and functional examination of the try-in and patient consent;
8. Laboratory final steps and denture finishing;
9. Delivery and final fit check of the finished dentures.

The analogue dentures were functionally, phonetically, and aesthetically accepted by the patient and have been evaluated as qualitative and satisfactory by the medical team. Therefore, the analogue dentures were considered as a satisfactory and desirable result that would be used as a reference further in the study.

**Fig. 1:**
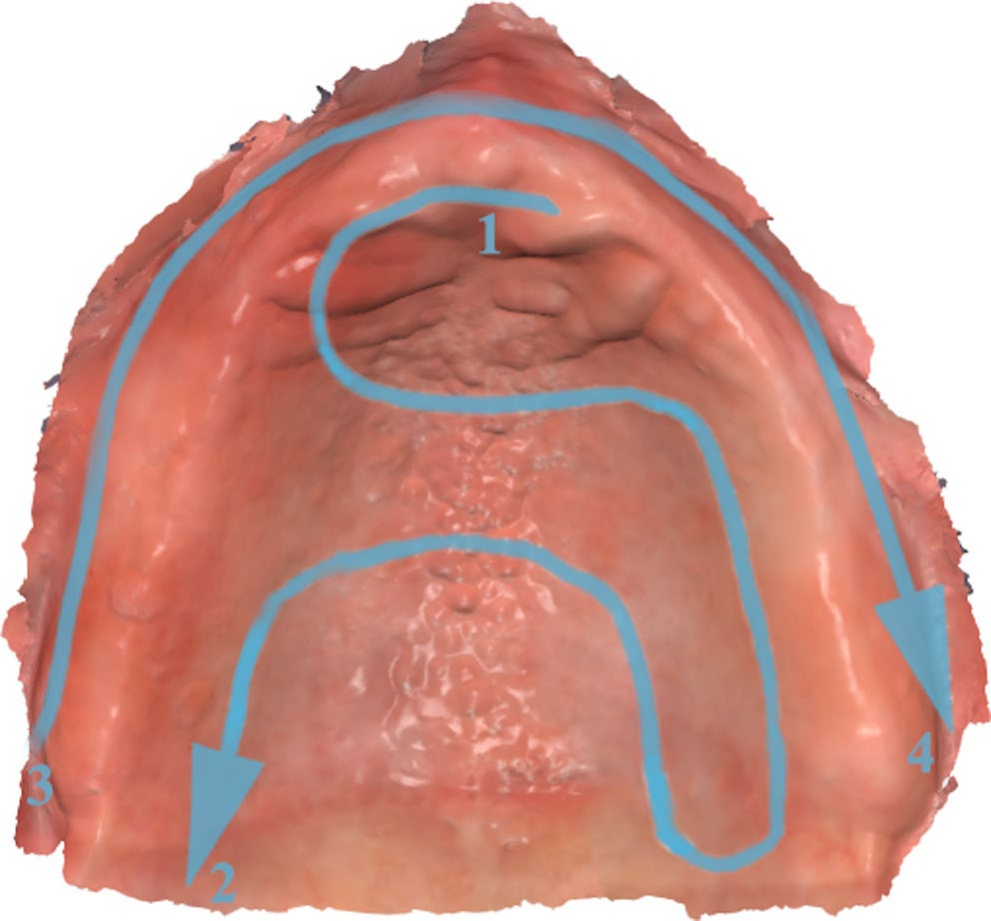
Edentulous area scanning pattern

The edentulous maxillary arch of each patient was scanned with an intraoral scanner (Medit I700, Medit, Seoul, South Korea) by the same experienced practitioner with over 20 years of experience in conventional complete denture fabrication as well as intraoral scanning and digital impressions, obtaining 3D meshes of the edentulous arches in STL (standard tessellation language) format. The intraoral scanner has been calibrated beforehand and between each clinical case according to the manufacturer’s indications. The scanning pattern was the same for all the executed scans striving to obtain the complete digital impression of the edentulous arch in around a constant time frame of 60 to 90 s for each case. The scanning pattern consisted in a palate-buccal technique starting on the median line near the incisive papilla, following the palatal rugae pattern on both sides towards the posterior vibrating line and finishing with the edentulous ridge and the buccal region (Fig. 1). This pattern was taken into consideration because of the palatal rugae presenting a good landmark for the intraoral scanner and due to the overall efficiency of generating the digital impression of the entire edentulous arch in a constant time frame [17].

**Fig. 2:**
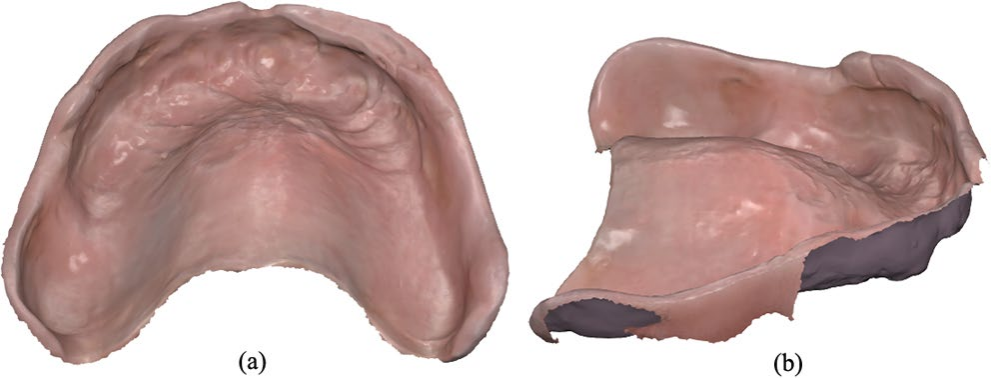
Intaglio of the delivered conventional denture generated as a 3D mesh after scanning. (**a**) Top view. (**b**) Side view

The intaglio of the delivered conventional denture was also digitized with the help of the same intraoral scanner (Medit i700) by using the “Additional Data” option from the “Stage Management” menu of the Medit Scan software to obtain a digital mesh in STL format (Fig. 2). The scanning protocol followed a similar pattern as the edentulous arch scanning protocol and was performed by the same experienced practitioner. Only the intaglio area was included in the scan as this was the only relevant area for the analyses in the present study.

The scan of the edentulous arch was imported into a dental design software (DentalCAD 3.0 Galway, Exocad GmbH, Darmstadt, Germany) to produce a digital denture base for each case following a complete digital workflow. All the dentures were digitally designed following the specific software steps by the same experienced dental technician that also produced the conventional protocol dentures.

**Fig. 3:**
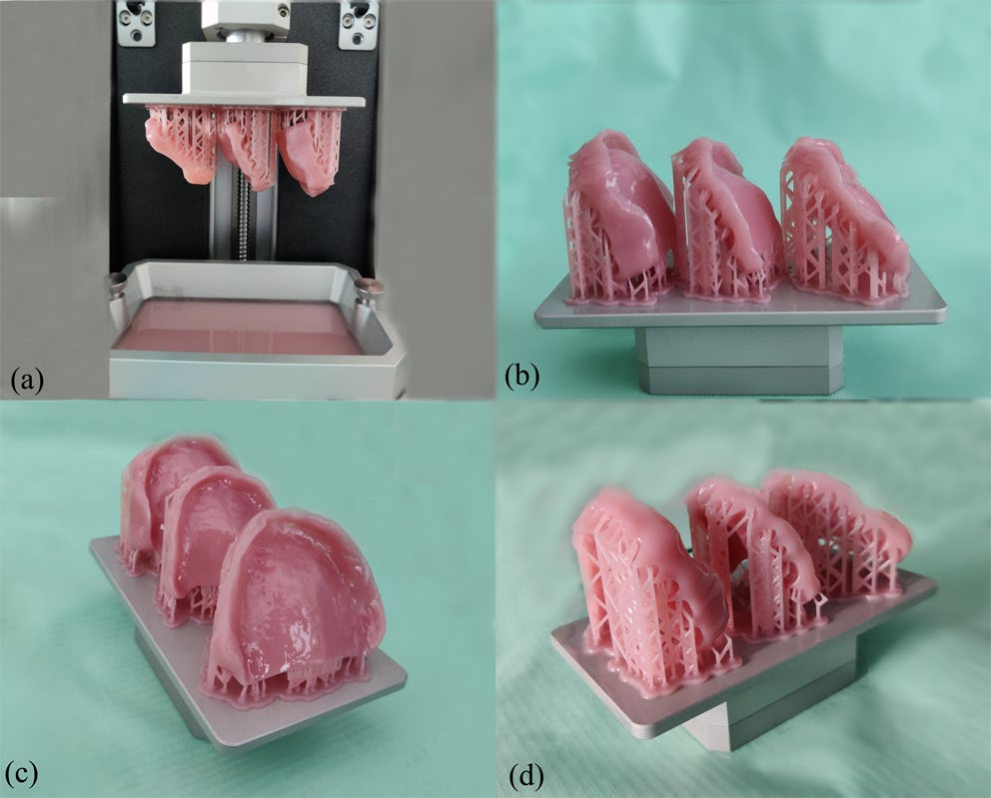
3D Printed denture bases with NextDent Denture 3D + biocompatible resin. (**a**) Denture bases inside the 3D printer. (**b**), (**c**), (**d**) 3D printed denture bases before the post-processing steps, viewed from different angles

**Fig. 4:**
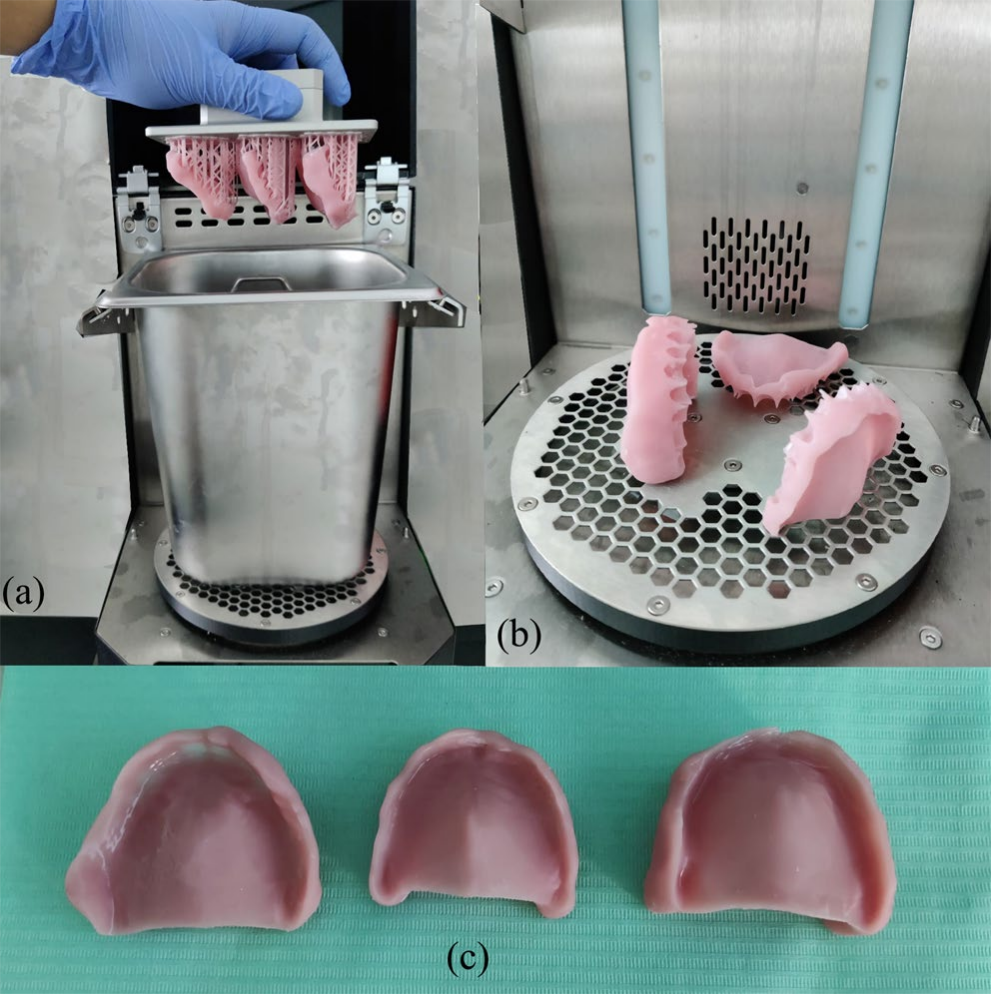
Post-processing of the 3D printed denture bases. (**a**) Washing in in ethanol (> 90%) bath for 5 min. (**b**) Removing the supports and post-curing under UV-light treatment for a total of 10 min. (**c**) Finished 3D printed denture bases

The finalized design of the denture bases was imported into the dedicated 3D printer software (Prusa Slicer 2.6.1) in order to add the supports, slice and prepare for printing. The denture bases have been angled so that the print supports do not reside on the intaglio surface to avoid producing any modifications on this surface. The denture bases were printed with the help of Prusa SL1 3D printer (Prusa Research, Prague, Czech Republic) using NextDent Denture 3D+ (NextDent, Soesterberg, Netherlands) biocompatible resin (Fig. 3). The denture base was angled at 45 degrees during the 3D printing process, positioning the intaglio surface facing upwards. The 3D printer was prior calibrated for the specified resin and printing conditions. The post-processing of the printed denture bases was carried out according to the resin manufacturer’s instructions including cleaning the printed parts for a total of five minutes in ethanol (> 90%), drying and post-curing under UV-light treatment for a total of 10 min. The post-processing protocol was executed with the use of the Prusa CW1S (Prusa Research, Prague, Czech Republic) curing and washing machine (max power of the UV LED: 52,8 W) (Fig. 4).

**Fig. 5:**
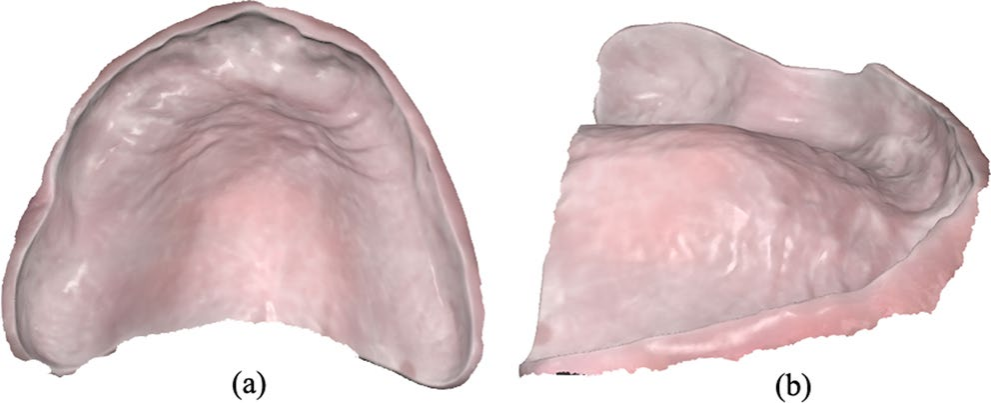
Intaglio of the printed denture base generated as a 3D mesh after scanning. (**a**) Top view. (**b**) Side View

The intaglio of the finished 3d printed denture bases were digitized with the help of the same intraoral scanner (Medit i700) (Fig. 5). The scanning protocol followed a similar pattern as the scanning of the conventional denture intaglio and was performed by the same experienced practitioner.

A complete metrology grade, quality control software equipped with powerful tools (Geomagic Control X, Version:16.0.2.16496, 3D Systems, Wilsonville, OR, USA) was used in order to assess the intaglio surface trueness of the printed denture bases and to compare them to the intaglio of the conventional dentures.

The analogue dentures were considered as the reference point for this part of the study, therefore, the intaglio surface of the 3D printed dentures would be compared to the intaglio surface of the analogue dentures to obtain the standard deviation value between those surfaces. The standard deviation value produced by the comparison would represent the intaglio surface trueness of the 3d printed denture, in other words how true is the 3d printed intaglio surface to the analogue intaglio surface which was deemed successful beforehand by the patient and the dental team.

**Fig. 6:**
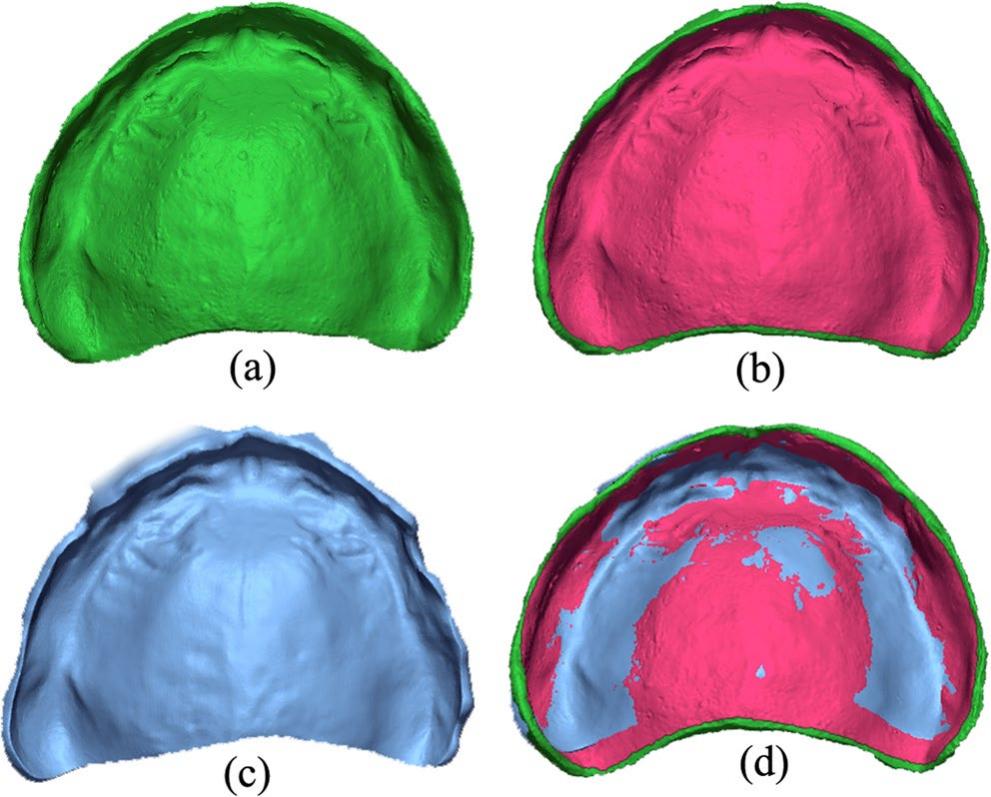
(**a**) 3D mesh of the analogue denture as reference data. (**b**) Isolated area of interest. (**c**) 3D mesh of the printed denture as measured data. (**d**) Precise alignment and overlapping of the measured data over the reference data

The STL file containing the 3D mesh of the analogue denture was imported into the metrology software and set as reference data (Fig. 6a). An area of interest, containing only the intaglio surface and excluding the periphery and marginal zone, was designated and isolated on the reference model (Fig. 6b) to facilitate data alignment and comparison. Only the surfaces residing inside the area of interest were analysed in the comparison process. The STL file containing the 3D mesh of the printed denture was imported into the metrology software and set as measurement data (Fig. 6c). The “initial alignment” function was used to superimpose the measure data over the reference data, followed by the “best fit alignment” function on the area of interest to obtain a precise overlapping (Fig. 6d).

**Fig. 7:**
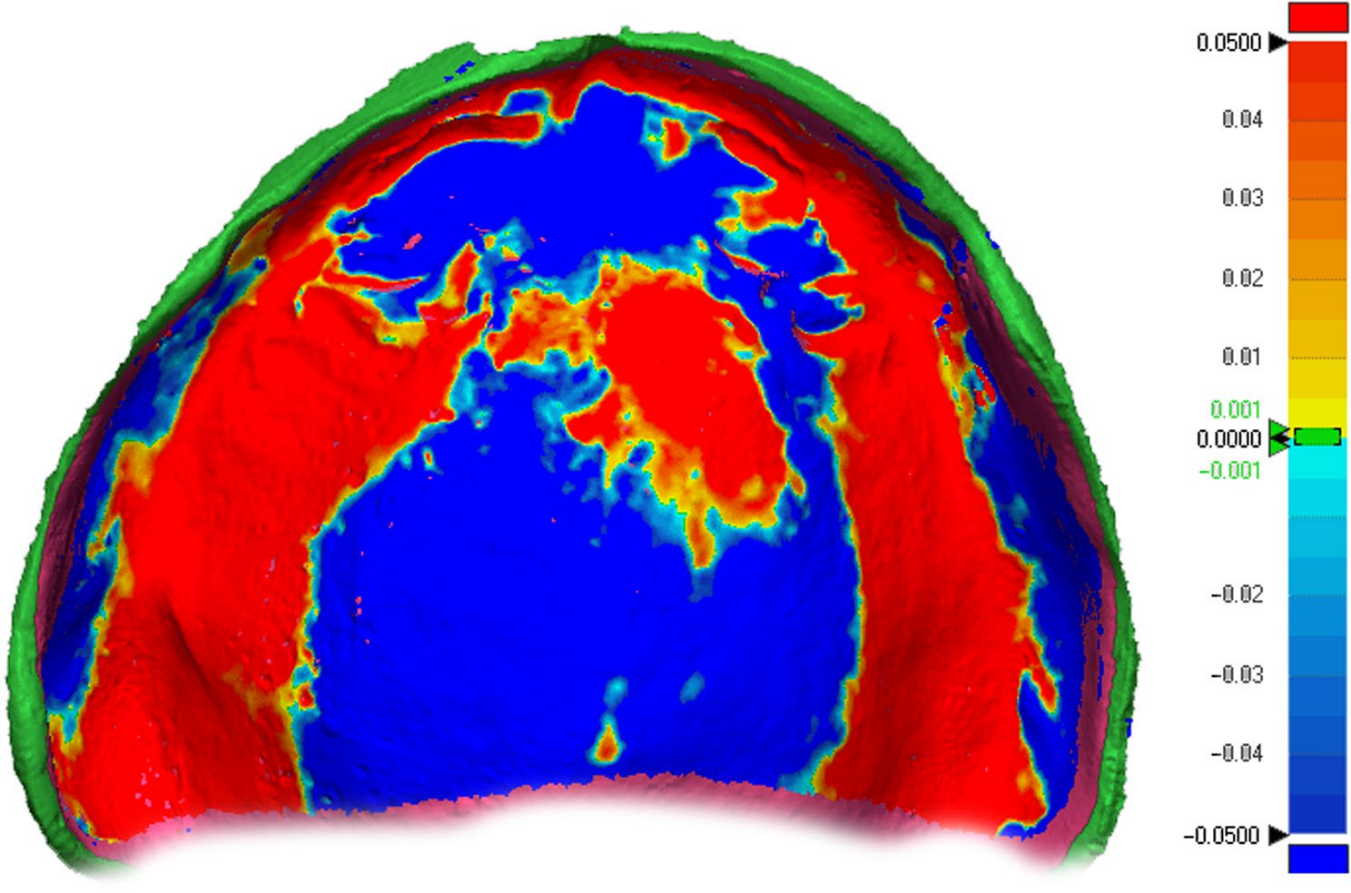
3D Comparison colour coded map displaying outward displacement in red and inward displacement in blue, both measured in millimetres

The “3D Compare” feature within the metrology software showcased the standard deviation outcomes by projecting all matched data points onto the reference dataset and the RMS (root mean square) values were collected from the report. Additionally, this function generated a color-coded map that visually represented the deviation patterns for the analysed surfaces within a range of ± 0.05 mm (50 μm) (Fig. 7). On the color-coded map, outward displacements were denoted by shades in the red spectrum, inward displacements were indicated in the blue spectrum, while the areas in green signified no deviation, as the difference was less than ± 1 μm.

The entire protocol was conducted for all the 15 subjects in order to obtain the standard deviation value of each case and to corroborate all the intaglio surface trueness values of the 3D printed dentures.

The same software (Geomagic Control X) was used to analyse the difference of intaglio surface trueness between analogue and 3D printed dentures in relation to the edentulous arch, following a similar protocol. The scans of the edentulous arch were considered as the reference point for this part of the study, therefore, the intaglio surface of the 3D printed dentures and conventional dentures respectively would be compared to the surface of the edentulous arch to obtain the standard deviation value between those surfaces for each comparison. The standard deviation value produced by each comparison would represent the intaglio surface trueness of the 3d printed denture and the intaglio surface trueness of the analogue denture respectively. In other words, revealing how true is the 3d printed intaglio surface to the scanned edentulous arch and how true is the analogue intaglio surface to the scanned edentulous arch.

**Fig. 8:**
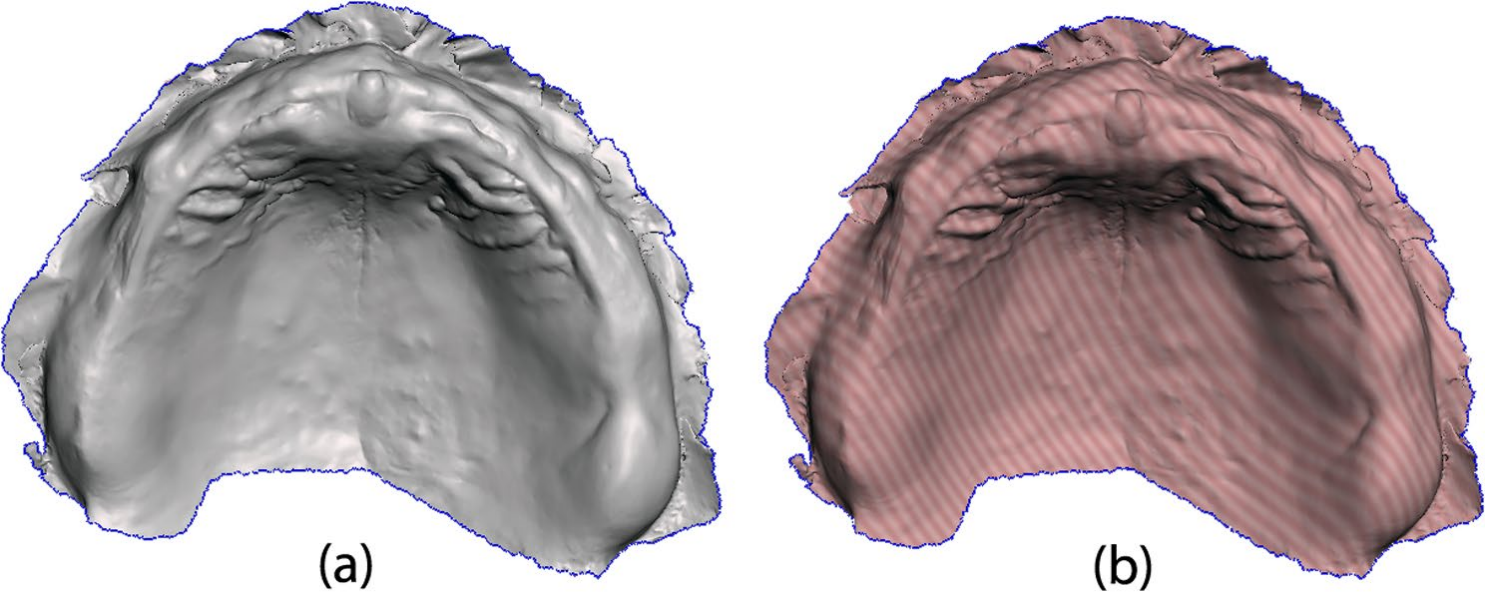
(**a**) 3D mesh of the arch mesh imported into Meshmixer software. (**b**) 3D mesh after flipping vertices with “Flip Normals” function

In order to achieve an accurate alignment, overlapping and 3D comparison, the metrology software must identify two similar surfaces, expecting to find convexities next to convexities and concavities next to concavities, not vice-versa. The edentulous arch scans present a positive landscape while the intaglio surface scans of the 3D printed and analogue dentures depict a negative landscape. For a correct alignment and comparison to take place inside the metrology software, the 3D meshes of the edentulous arch files were inverted with the help of Autodesk Meshmixer software (Version 3.5, Autodesk, San Rafael, CA, USA) by using the “Flip Normals” function (Fig. 8).

**Fig. 9:**
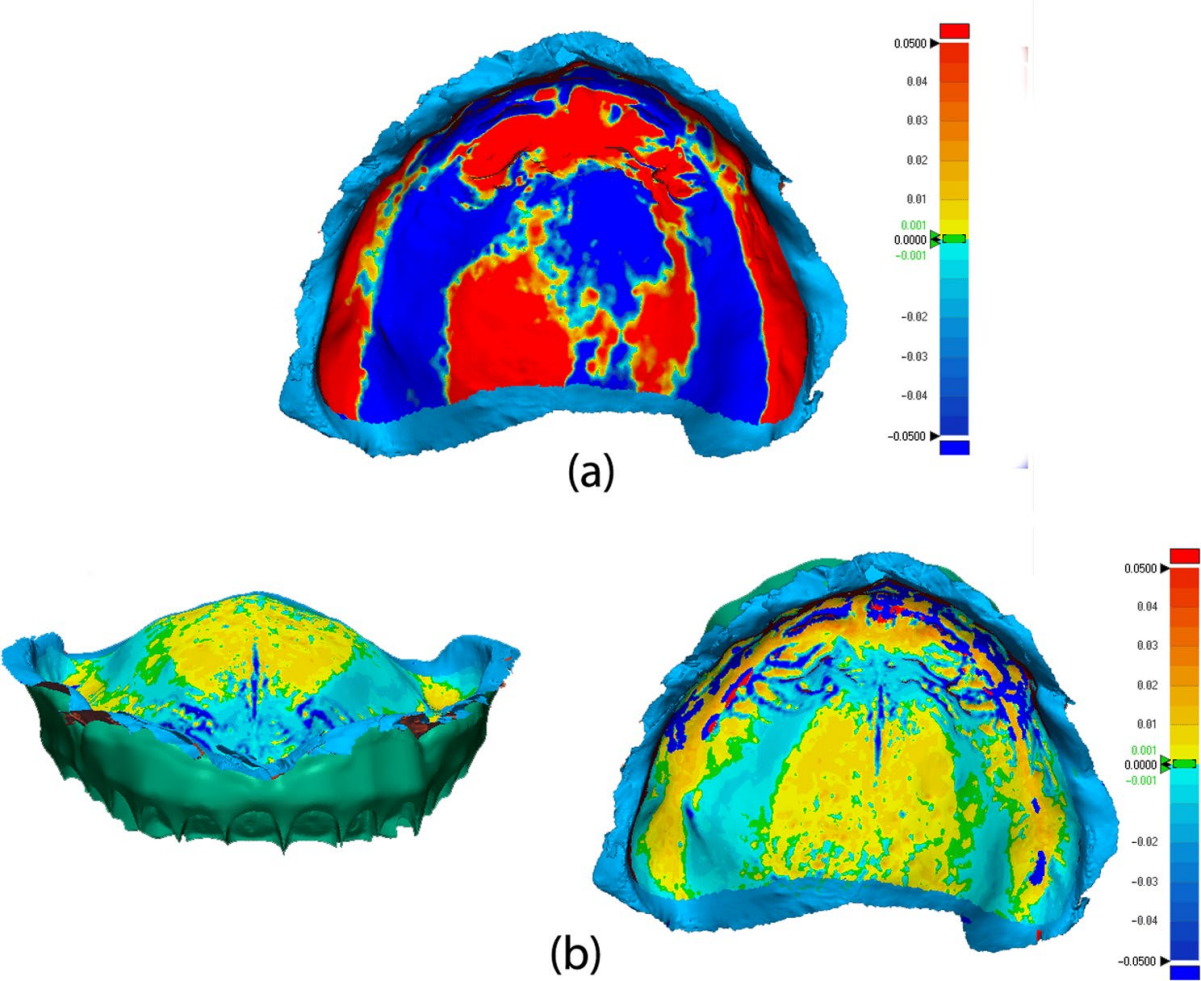
(**a**) 3D Comparison colour coded map of the analogue denture intaglio surface, displaying outward displacement in red and inward displacement in blue, measured in millimetres. (**b**) 3D Comparison colour coded map of the 3D printed denture intaglio surface

The STL file containing the 3D mesh of the inverted edentulous arch was imported into the metrology software and set as reference data. An area of interest, containing only the intaglio surface was designated and isolated on the reference model. The STL files of the 3D printed denture and the analogue denture were imported into the metrology software and set as measured data. “Initial alignment”, “best fit alignment” and “3d compare” functions were executed for each measured data to obtain the intaglio surface trueness values for the printed denture and analogue denture respectively. The standard deviation outcomes alongside the color-coded maps were generated for each analysed subject (Fig. 9). The entire protocol was conducted for all 15 cases and the values were corroborated to further analyse the data.

The collected trueness values were imported into the MedCalc statistical software (MedCalc Software Ltd, Ostend, Belgium) for the purpose of performing the statistical analysis. An initial step involved subjecting the entire dataset to the Kolmogorov-Smirnov test to assess its normality, revealing that the values did not adhere to a parametric distribution. Subsequently, the dataset was subjected to Kruskal-Wallis test analysis, along with the execution of a post-hoc analysis using the Conover test. The significance level for this analysis was established at α = 0.05.

## Results

The standard deviation values of the intaglio surface of 3D-printed dentures in relation to the analogue dentures are presented in Table 1.

**Table 1:** Trueness of 3D-printed denture intaglio when compared to the analogue denture intaglio of each sample, presented in microns alongside the median and the interquartile range (IQR) of the values

Sample	Std. Dev.	Summary values
S1	177.8 μm	Median = 183.8 μm
S2	223.7 μm	IQR = 46.2 μm
S3	215.4 μm	
S4	332.4 μm	
S5	221.7 μm	
S6	175.5 μm	
S7	205.6 μm	
S8	229.9 μm	
S9	183.8 μm	
S10	142.6 μm	
S11	180.7 μm	
S12	176.6 μm	
S13	116.3 μm	
S14	170.1 μm	
S15	186.3 μm	

The intaglio surface of the 3D-printed dentures displayed an overall 184 μm deviation from the intaglio surface of the analogue dentures with the highest value sample displaying 332 μm deviation, showing the least trueness, and the lowest value sample displaying 116 μm deviation, showing the best trueness.

The intaglio surface trueness values of the 3D-printed dentures and the analogue dentures respectively in relation to the intraoral edentulous area scan are presented in Table 2.

**Table 2:** Median and interquartile range (IQR) of the intaglio surface trueness values of the 3D-printed denture and the analogue denture respectively

Group	Median	IQR
3D-Printed Denture	176.9 μm	67.4 μm
Analogue Denture	518.7 μm	206.6 μm

**Fig. 10:**
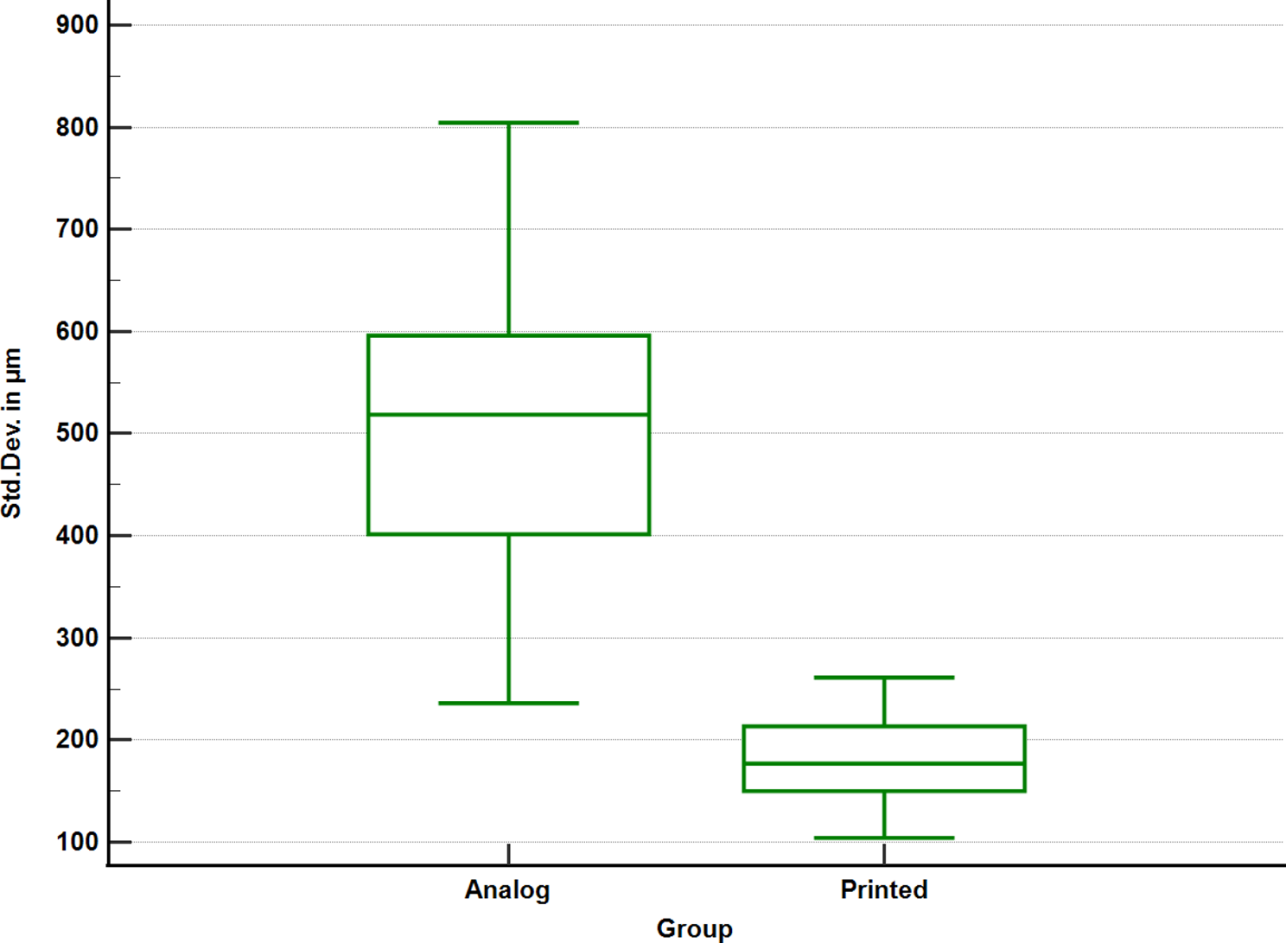
Boxplot presenting the intaglio surface trueness values of the analogue denture group and the 3d-printed denture group

The analysis indicated that there is a statistically significant difference between the trueness values of the 3D-printed denture group and the analogue denture group (*p* < 0.0001). The 3D-printed denture group displayed better trueness values with a median of 176.9 μm, indicating a better intaglio surface fit on the edentulous arch. The trueness values of the analogue denture group displayed an overall 342 μm increase in deviation compared to the 3D-printed denture group (Fig. 10).

## Discussion

The treatment of edentulous patients may follow the three existing paths for manufacturing the complete dentures: the conventional method, the modified conventional method with the scanning of the conventional impression and the fully digital approach with the direct scan of the edentulous arches. Additionally, CAD/CAM dentures can be manufactured either from milling the denture base from a resin block or alternatively, through the additive method of printing the denture base [18, 19].

There have been reported mixed results regarding the fit and work efficiency between conventional milled and printed complete dentures.

One study investigated the difference in number of visits and remake rate of conventionally fabricated and digitally fabricated complete dentures. The results of the study showed that the digitally protocol required less appointments from start to finish, and fewer postoperative visits than conventionally fabricated dentures [20].

Another study evaluated denture base adaptation fabricated using conventional, subtractive, and additive technologies. The authors concluded that milled denture bases showed better adaptation than 3D printed or conventionally fabricated denture bases for both maxillary and mandibular arches [21].

Other authors investigated the patient’s satisfaction when using conventionally vs. digitally fabricated dentures. Ohara K, et al. reported that patient satisfaction with conventional dentures was superior in terms of phonetics, ease of cleaning, stability, comfort, but the investigated groups showed no significant differences in the other outcomes [22]. However, another study showed no difference in patient preference between milled, printed or conventional dentures [23].

Other recent studies were able to digitally asses with success the total deviation values of different impression techniques in free end saddle partially edentulous patients by implying the same method with the help of Geomagic Contol X metrology software [24]. The same software proved as a reliable tool of comparing digital with analogue protocols in a recent study analyzing the accuracy of digital auricular impression using intraoral scanner versus conventional impression technique for ear rehabilitation [25].

The present study investigated whether 3D printed dentures developed with a fully digital workflow can produce an intaglio surface similar to the already clinically accepted analogue dentures. The trueness median of the analysed 3D printed samples displayed a value of 184 μm deviation from the intaglio surface of the analogue dentures. Considering the large surface of the inspected area, the deviation value should be clinically acceptable. Therefore, the 3D-printed dentures should behave reasonably well, just as the analogue dentures, when accounting for the intaglio surface. These values only show the discrepancy between the intaglio surface of the two groups of dentures and do not provide indication of fidelity adaptation on the edentulous arch. The printed denture bases have been tried in vivo on edentulous maxillary arches and have successfully shown optimal retention. However, these aspects are not presented in the current study, since it is was not possible to objectively quantify the denture retention and other aspects observed during the try-in of the printed denture bases.

One of the novelties of the present study lies in the method of analysing the trueness of the intaglio surfaces of printed and analogue dentures in relation to the edentulous arch in such a way as to indicate the adaptation of these surfaces on the edentulous arch. The intraoral scans of the edentulous arches were inverted in the dedicated software to provide a feasible method of aligning, overlapping and comparing the intaglio surfaces of the denture groups to the edentulous. Similar studies talk about the intaglio surface trueness of 3d printed and milled denture bases, but the analysis method of the cited study compares the scanned surface of the produced dentures to the CAD design mesh of the dentures, thus proving only how accurate is the printing or milling process and do not provide insight on the fit of the finished printed or milled denture on the actual edentulous arch [26].

In order to indicate which of the two groups will have a better fit on the edentulous arch, the trueness of each group (3d-printed and analogue) was analysed in relation to the edentulous arch. The 3D-printed denture group displayed better trueness values indicating a better intaglio surface fit on the edentulous arch while the trueness values of the analogue denture group displayed an overall 342 μm increase in deviation. Provided that the analogue dentures were already considered to have a clinically acceptable fit on the edentulous arches, the results denote that the 3D-printed dentures show an even better and improved fit of the analysed intaglio surface. Yiyang Wang et al. showed that the outcomes of their in vivo studies revealed a trueness range spanning from 40 to 1380 μm, while the precision results were not provided [27].

This outcome was to be expected due to a combination of factors influencing the final accuracy of the intaglio surfaces of the prostheses. The analogue dentures go through several error-prone clinical and laboratory stages that rely on the accuracy of the impression material, the accuracy of the plaster that is used to cast the models as well as the accuracy and potential errors during the fabrication of the denture bases and their finishing protocols [28, 29]. There is also a strong component of human error during all these processes, as all steps are carried out analogically and depend on the experience and precision of the operator. The fit of 3D printed dentures is influenced by the accuracy of the intraoral scanner, the accuracy and potential errors of the resin and printing process as well as the post-processing steps [30–33]. Emphasis must be placed on the fact that the dental cad software is able to produce a surface that better emulates the scanned edentulous arch thus providing a better surface trueness of the denture areas that will be in contact with the clinical soft tissue.

The clinical cases for this study were chosen so that the edentulous maxillary arch was suitable for the mucostatic technique. Nevertheless, the analogue dentures analysed in this study were adapted and validated so that they met the proper functional and aesthetic requirements.

A recent study analyzing the trueness of intraoral scanning of edentulous arches had similar findings concluding that the intraoral scanning protocol for edentulous arches showed statistically significant differences with a large effect size compared to the cast digitization protocol, however achieving a functional shape for the dynamic mobile areas of the peripheral borders remained challenging [34].

An essential aspect of the success of a denture is based on the accurate recording of the mobility of the periphery of the edentulous arches, guided by both the clinician and the orofacial muscular system [8, 9].

This article could have been improved by analyzing, through objective and quantifiable methods, the degree of retention of the printed denture bases. A systematic review in 2023 evaluating the clinical and laboratory procedures for digital complete dentures stated that making a border-molded impression remains the preferred method for better retention, and trial denture try-in is still advisable to improve the protocol of definitive digital complete dentures [35].

One limitation of direct intraoral scanning of soft tissues is the fact that it cannot adequately record mucosal elasticity and resilience. Intraoral scanners have difficulties when dealing with mobile intraoral tissues, making impression of the periphery of the prosthetic field quite difficult and questionable in certain clinical conditions and cases [27]. An in-vivo study showed that the digitization of edentulous jaws using intraoral scanning seemed to be a viable approach, but it struggled to accurately replicate the peripheral tissues [36]. It should be mentioned that the adhesion of a maxillary denture depends on the intimacy of the intaglio which can compensate for the deficiencies in the adhesion force produced at the edges of the dentures. The peripheral tissue area was not analysed in the present study and future studies are required to determine these aspects regarding the clinical success of 3D printed dentures produced by following a fully digital workflow.

Advances in intraoral scanner technology that would allow accurate impressions of the intraoral mobile mucosa and the peripheral tissues would allow the production of clinically successful complete dentures by fully digital protocol. Further studies must be conducted on this topic to better evaluate the clinical success of manufacturing complete dentures produced by a fully digital workflow.

## Conclusions

Based on the findings of this clinical study, the following conclusion can be drawn:

1. The 3D-printed denture bases presented favorable trueness values of the intaglio surface compared to the clinically accepted conventional dentures.
2. Significant differences were found supporting 3D-printed denture bases over analogue denture bases in terms of intaglio surface trueness and fit on the edentulous arch, implying a potential increase in accuracy and fit in the critical intaglio region.
3. The findings suggest that digital methods could improve the precision and reliability of complete denture production, especially in replicating the intaglio surface of the denture base .

## Data Availability

The datasets used and/or analysed during the current study are available from the corresponding author on reasonable request, at andrei.faur@umft.ro, +40744673091, Dr. Faur Andrei-Bogdan.
